# MicroRNA‐876‐5p inhibits cell proliferation, migration and invasion by targeting c‐Met in osteosarcoma

**DOI:** 10.1111/jcmm.14217

**Published:** 2019-02-17

**Authors:** Weixin Xie, Jie Xiao, Tao Wang, Dongmei Zhang, Zhanchun Li

**Affiliations:** ^1^ Department of Orthopaedic Surgery, School of Medicine Renji Hospital, Shanghai Jiaotong University Shanghai China; ^2^ Department of Anesthesiology, School of Medicine Renji Hospital, Shanghai Jiaotong University Shanghai China; ^3^ School of Biomedical Sciences, Center for Orthopaedic Translational Research University of Western Australia Nedlands Australia; ^4^ Clinical Medical Research Center The Second Affiliated Hospital of Nantong University Nantong Jiangsu Province China

**Keywords:** c‐Met, metastasis, miR‐876‐5p, osteosarcoma, proliferation

## Abstract

Recently, aberrant expression of miR‐876‐5p has been reported to participate in the progression of several human cancers. However, the expression and function of miR‐876‐5p in osteosarcoma (OS) are still unknown. Here, we found that the expression of miR‐876‐5p was significantly down‐regulated in OS tissues compared to para‐cancerous tissues. Clinical association analysis indicated that underexpression of miR‐876‐5p was positively correlated with advanced clinical stage and poor differentiation. More importantly, OS patients with low miR‐876‐5p level had a significant shorter overall survival compared to miR‐876‐5p high‐expressing patients. In addition, gain‐ and loss‐of‐function experiments demonstrated that miR‐876‐5p restoration suppressed whereas miR‐876‐5p knockdown promoted cell proliferation, migration and invasion in both U2OS and MG63 cells. In vivo studies revealed that miR‐876‐5p overexpression inhibited tumour growth of OS in mice. Mechanistically, miR‐876‐5p reduced c‐Met abundance in OS cells and inversely correlated c‐Met expression in OS tissues. Herein, c‐Met was recognized as a direct target of miR‐876‐5p using luciferase reporter assay. Notably, c‐Met restoration rescued miR‐876‐5p attenuated the proliferation, migration and invasion of OS cells. In conclusion, these findings indicate that miR‐876‐5p may be used as a potential therapeutic target and promising biomarker for the diagnosis and prognosis of OS.

## 1 INTRODUCTION

Osteosarcoma (OS) is a highly malignant bone tumour that mainly occurs in adolescents and young adults.^1^ Although the therapy of OS has improved in the past decade, the prognosis of OS is still very poor. The main reason for poor prognosis of OS patients is the metastases after surgical resection and chemotherapy.^2^, ^3^ The pathogenesis, progression and prognosis of OS are regulated by many tumour‐related signalling pathways.^3^ However, the molecular mechanisms involved in the formation and development of OS are limitedly explored. Thus, it is urgent to elucidate the potential mechanism and seek new therapeutic targets for OS treatment.

MicroRNAs (miRNAs) are proved to regulate gene expression by binding to the 3′UTR of their target mRNAs.^4^ Recent studies have found that miRNAs are important cancer biomarkers and play a key role in cancer cell growth and metastasis.^5^, ^6^ Tumour‐associated miRNAs can function as both tumour suppressors and oncogenes, which depend on the biological function of their target genes.^9^, ^10^ For instance, miR‐506‐3p functions as a tumour suppressor by suppressing the proliferation and metastasis of OS cells via targeting RAB3D.^11^ MiR‐613 inhibits OS cell proliferation and migration, and induces apoptosis by targeting chemokine receptor 4 (CXCR4).^12^ While, our previous study reports that miR‐92a is overexpressed in OS tissues and facilitates tumour growth of OS via suppressing phosphatase and tensin homologue (PTEN) and AKT signalling pathway.^13^ Recently, A new tumour‐suppressive miR‐876‐5p has been found to play important roles in a variety of tumours including lung cancer,^14^ oesophageal squamous cell carcinoma (ESCC),^15^ hepatocellular carcinoma (HCC)^16^, ^17^ and head and neck squamous cell carcinoma (HNSCC).^18^ MiR‐876‐5p inhibits the epithelial‐mesenchymal transition (EMT) and tumour metastasis in lung cancer, HCC and HNSCC.^14^, ^16^, ^18^ Moreover, miR‐876‐5p suppresses tumour growth and metastasis of ESCC by targeting tumour antigen MAGE‐A family.^15^ However, the biological function and potential molecular mechanisms of miR‐876‐5p in human OS are unclear.

In this study, we first determined the expression of miR‐876‐5p between OS and matched para‐cancerous tissues. Then, the clinical significance, biological functions and related molecular mechanisms of miR‐876‐5p were investigated. In conclusion, our results suggest that miR‐876‐5p may be an effective therapeutic target for OS patients.

## 2 MATERIALS AND METHODS

### 2.1 Clinical specimens

Sixty‐eight OS tissues and matched para‐cancerous tissues were collected from Renji Hospital, School of Medicine, Shanghai Jiaotong University. None of the OS patients received chemotherapy or radiotherapy before surgery. The collected specimens were obtained with the informed consent of the patients or the patient's families. The collected OS tissue and para‐cancerous tissues were stored immediately in liquid nitrogen for subsequent analysis. The protocols and consents were approved by the Ethics Committee of Shanghai Jiaotong University and complied with the Declaration of Helsinki.

### 2.2 Cell culture and transfection

The human OS cell lines U2OS and MG63 were saved in our laboratory.^13^ OS cells were cultured in DMEM medium (Hyclone, Logan, UT) containing 10% Fetal bovine serum (FBS, Gibco, Grand Island, NY), streptomycin (100 µg/mL; Sigma‐Aldrich, St. Louis, MO) and penicillin (100 U/mL, Sigma‐Aldrich) at 37°C in a humidified atmosphere comprising 5% CO_2_.

Lentiviral vector‐mediated precursor miR‐876‐5p and negative control were obtained from Sigma‐Aldrich Co. LLC. MiR‐876‐5p inhibitors in lentiviral vectors and control inhibitors were purchased from Geneopenia (Guangzhou, China). OS cells were infected with lentiviruses in the presence of Polybrene (8 ng/mL, Sigma‐Aldrich). After overnight incubation, the virion‐containing culture supernatant was removed and replaced with fresh virus‐free medium. Full‐length sequences of c‐Met cDNA were PCR amplified and subcloned into the pcDNA3.1 plasmid (Invitrogen, Carlsbad, CA). Vectors were transfected into OS cells using Lipofectamine 2000 Reagent (Life Technology, Thermo Fisher Scientific, Waltham, MA).

### 2.3 Quantitative real‐time PCR

Total RNA was extracted from all OS tissues and cells using TRIZON reagent (Thermo Fisher Scientific, Inc) according to the manufacturer's protocol. cDNA was synthesized according to the instructions of the 5X‐All‐In‐One RT Master MIX kit (Applied Biological Materials, Inc, Richmond, BC, Canada). The synthesized cDNA was subjected to real‐time PCR using an All‐in‐One miRNA qPCR Detection kit (GeneCopoeia, Rockville, MD) for miR‐876‐5p expression and SYBR^®^ Premix Ex Taq^™^ II (Takara, Dalian, China) for c‐Met mRNA expression, respectively, in an ABI PRISM 7300 Sequence Detection system (Applied Biosystems, Foster City, CA). U6 and GAPDH were used as standardized internal references and calculated using the 2^−ΔΔCt^method for relative quantification. The sequences of all primers are shown in Table S1.

### 2.4 Cell Counting Kit‐8 assays

U2OS and MG63 cells were first seeded into 96‐well tissue culture plates (3 × 10^3^ cells per well). The detection was performed at 0, 24, 48 and 72 hours. After adding 10 µL of Cell Counting Kit‐8 (CCK8) reagent (Dojindo Molecular Technologies, Inc, Kumamoto, Japan) per well and incubating for 2 hours, the absorbance at 450 nm was measured using a Multiscan FC plate reader and analysed with SkanIt for Multiscan FC software (Thermo Scientific).

### 2.5 Cell proliferation analysis

The 5‐ethynyl‐2'‐deoxyuridine (EdU) proliferation assay was used to measure cell proliferation. We added 0.1 mL of 50 μmol/L EdU (RiboBio Biotechnology, Guangzhou, China) into each well of 500 mL medium for 2 hours. Then, cells were fixed with 4% polyformaldehyde in PBS at room temperature for 30 minutes and subsequently incubated with Apollo staining solution and Hoechst 33342 for 30 minutes. Fluorescence microscopy was performed in five randomly selected fields with an Zeiss fluorescence photomicroscope (Carl Zeiss, Oberkochen, Germany) to analyse proliferation rates.

### 2.6 Cell migration and invasion assay

The migration and invasion ability of U2OS and MG63 cells were assessed using 6.5‐mm transwell chambers with a pore size of 8 μm (Costar, Corning, NY). Cells (2 × 10^5^) were suspended in 100 μL serum‐free medium and seeded into the upper chamber or precoated with 80 μL of Matrigel solution (BD, Franklin Lakes, NJ) for cell invasion assay. The lower chamber was filled with 600 μL of 10% FBS medium. After incubation for 48 hours, cells that had migrated or invaded to the lower side of the membrane were fixed and stained with 10% Giemsa. Five random fields were chosen to count and take photos under a microscope.

### 2.7 Western blotting analysis

Total cellular protein was extracted using Radio‐Immunoprecipitation Assay (RIPA) lysis buffer (25 mmol/L Tris‐HCl + 150 mmol/L NaCl + 1% NP‐40 + 1% sodium deoxycholate + 0.1% SDS, pH 7.6) supplemented with 1% protease inhibitors (100×; Roche, Indianapolis, IN) and phenylmethanesulfonyl fluoride (PMSF, 100 mmol/L, Sigma Chemical Co., St. Louis, MO). The protein concentration is quantified by bicinchoninic acid (BCA) protein assay kit (Pierce, Rockford, IL). Equal amount of protein from cell lysates was separated by 10% SDS‐PAGE gels and transferred to PVDF membranes (Millipore Corp., Billerica, MA). After being blocked with 5% fat‐free milk, the membranes were incubated with the following primary antibodies: c‐Met (1:1000; Abcam, Cambridge, MA) and Glyceraldehyde‐3‐Phosphate Dehydrogenase (GAPDH; 1:10000; Abcam; the loading control) at room temperature for 3 hours, and then incubated with sheep‐anti‐mouse or donkey‐anti‐rabbit horseradish peroxidase‐conjugated (HRP) antibody (GENXA931‐1ML and GENA934‐1ML, Sigma‐Aldrich). Blots were developed using Immobilon Forte Western HRP substrate (Millipore) and semi‐quantified by ImageJ software (1.46; National Institutes of Health, Bethesda, MD).

### 2.8 Tumour xenograft assay

Female athymic BALB/c nude mice (4‐6 weeks old) were obtained from Charles River Laboratories (Beijing, China). All animal experiments were approved by the Institutional Animal Care and Use Committee of Shanghai Jiaotong University. Scrambled control or precursor miR‐876‐5p‐infected U2OS cells were suspended in PBS and the cell concentration was adjusted to 1 × 10^8^/mL. Each mouse was subcutaneously injected with 100 μL cell suspension (six mice per group) in the right flank, and the tumour size was measured every 4 days and calculated as *a* × *b* ^2^/2 mm^3^ (*a*, long diameter; *b*, short diameter). Thirty days later, mice were killed after by cervical dislocation under anaesthesia, and the tumours were separated and processed for immunohistochemistry (IHC) to detect Ki‐67 expression.^19^

### 2.9 Target identification and dual luciferase assay

To identify the target, miR‐876‐5p was subjected to the online platform starBase V3.0.^20^ For the luciferase reporter assay, the binding sites of the wild‐type (wt) and the mutated (mt) c‐Met 3′‐UTR were cloned into the downstream region of the luciferase gene in the PGL3‐REPORT luciferase vector (Invitrogen). The HEK293T cells were then subjected to cotransfection with the wt or mt PGL3‐c‐Met‐3′UTR vectors and precursor miR‐876‐5p or miR‐876‐5p inhibitors. Finally, the luciferase activity was determined using a Luciferase Reporter Assay kit (Promega Corporation, Madison, WI) as per the manufacturer's protocol, and Renilla luciferase activity was used to normalize the data.

### 2.10 Statistical analysis

All data in the experiments were analysed statistically using GraphPad Prism 6.0 Software (GraphPad Inc, San Diego, CA). All experiments were repeated at least three times and the data were expressed as mean ± SD. Statistical differences were performed with Student's *t* test (two groups) or ANOVA followed by Tukey's test (three groups) as appropriate. The clinical association analysis was analysed with Chi‐square test or Fisher's exact test. The correlation analysis was analysed using the Pearson correlation test. Survival curves were evaluated using the Kaplan‐Meier method and differences between survival curves were tested by the log‐rank test. *P* < 0.05 was considered statistically significant.

## 3 RESULTS

### 3.1 miR‐876‐5p is down‐regulated in OS tissues

**Figure 1:**
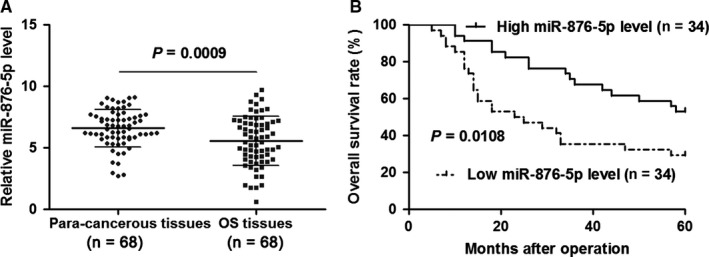
The expression and prognostic significance of miR‐876‐5p in osteosarcoma (OS). A, The relative level of miR‐876‐5p in 68 pairs of OS and matched para‐cancerous tissues was quantified by quantitative real‐time PCR *P* = 0.0009 by *t* test. B, OS patients were divided into two subgroups (low/high miR‐876‐5p level, n = 34 per group) using the median level of miR‐876‐5p as a cut‐off value. Low miR‐876‐5p level in OS tissues predicted poor prognosis of OS patients. *P* = 0.0108 by log‐rank test

In order to investigate the clinical significance of miR‐876‐5p in OS, we detected the expression of miR‐876‐5p using quantitative real‐time PCR (qRT‐PCR). As shown in Figure 1A, the expression levels of miR‐876‐5p were detected in 68 pairs of OS and matched para‐cancerous tissues. The results revealed that miR‐876‐5p expression was significantly down‐regulated in OS tissue samples compared to para‐cancerous tissues (*P* = 0.0009, Figure 1A). OS patients were divided into two subgroups (low/high miR‐876‐5p level) using the median level of miR‐876‐5p as a cut‐off value. As shown in Table 1, the low expression of miR‐876‐5p was positively correlated with advanced clinical stage (*P* = 0.026) and poor differentiation (*P* = 0.006). Moreover, OS patients with low miR‐876‐5p level had a significant shorter overall survival compared to cases with high miR‐876‐5p level (*P* = 0.0108, Figure 1B). Thus, miR‐876‐5p expression may be a promising prognostic marker for OS patients.

**Table 1:** Correlation between the clinicopathologic characteristics and miR‐876‐5p expression in osteosarcoma (n = 68)

Clinicopathologic features	n	miR‐876‐5p expression		*P*
		High	Low	
Gender				
Male	40	19	21	0.622
Female	28	15	13	
Age (y)				
<24	49	26	23	0.438
≥25	19	18	11	
Clinical stage				
I	27	18	9	0.026*
II + III	41	16	25	
T classification				
T1	26	16	10	0.134
T2	42	18	24	
M classification				
M0	61	32	29	0.427
M1	7	2	5	
Histology				
Conventional osteosarcoma	58	28	30	0.493
Others	10	6	4	
Histological differentiation				
G1	27	19	8	0.006*
G2	41	15	26	

* Statistically significant.

### 3.2 miR‐876‐5p inhibits OS cell proliferation, migration and invasion

**Figure 2:**
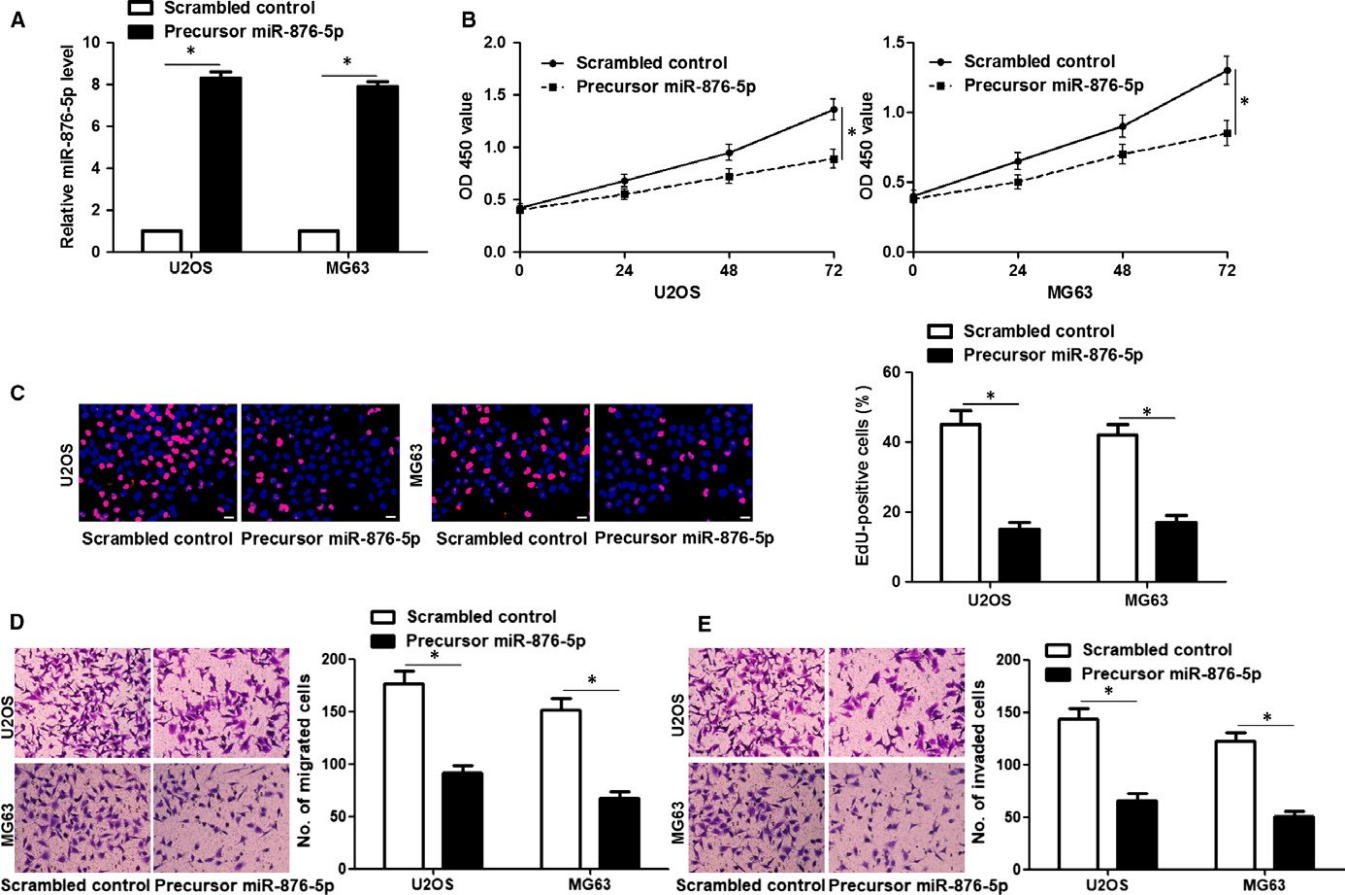
miR‐876‐5p restoration inhibits the proliferation, migration and invasion of osteosarcoma (OS) cells. A, U2OS and MG63 cells that were transfected with lentivirus‐mediated scrambled control or precursor miR‐876‐5p were detected by qRT‐PCR for miR‐876‐5p expression. n = three independent repeats, *P < 0.05 by *t* test. B, Cell Counting Kit‐8 assay investigated that miR‐876‐5p overexpression inhibited the viability of OS cells. n = three independent repeats, *P < 0.05 by ANOVA. C, 5‐ethynyl‐2'‐deoxyuridine (EdU) assay revealed that miR‐876‐5p restoration suppressed the proliferation of OS cells. Scale bar: 20 μm, n = three independent repeats, *P < 0.05 by *t* test. (D,E) Transwell assay indicated that the migration and invasion ability of cells were restrained by miR‐876‐5p overexpression in both U2OS and MG63 cells. n = three independent repeats, *P < 0.05 by *t* test

**Figure 3:**
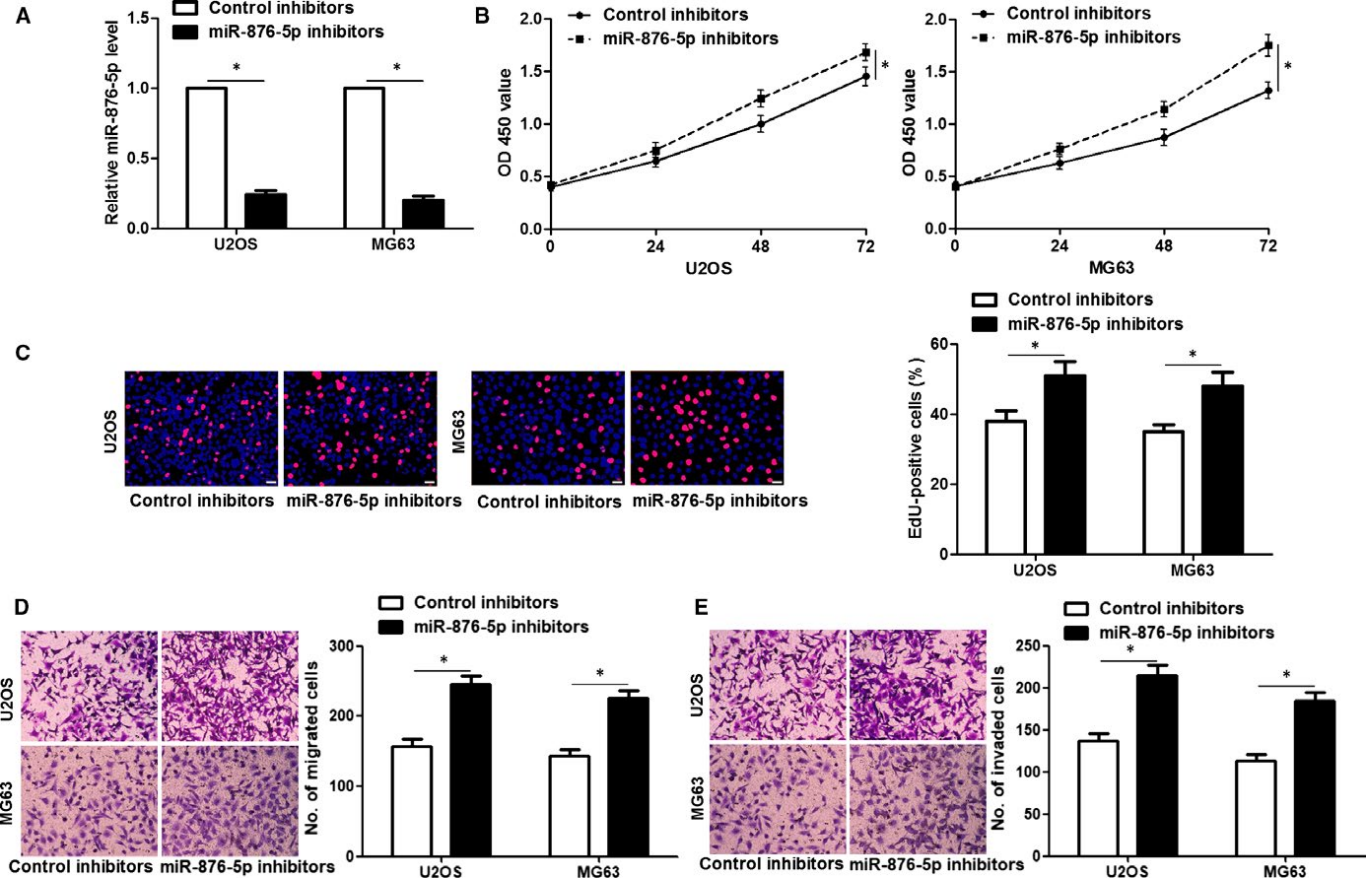
miR‐876‐5p knockdown promotes osteosarcoma (OS) cell proliferation, migration and invasion. A, U2OS and MG63 cells that were transfected with lentivirus‐mediated control inhibitors or miR‐876‐5p inhibitors were detected by qRT‐PCR for miR‐876‐5p expression. n = three independent repeats, *P < 0.05 by *t* test. B, Cell Counting Kit‐8 assay investigated that miR‐876‐5p knockdown promoted the viability of OS cells. n = three independent repeats, *P < 0.05 by ANOVA. C, 5‐ethynyl‐2'‐deoxyuridine (EdU) assay revealed that miR‐876‐5p knockdown facilitated the proliferation of OS cells. Scale bar: 20 μm, n = three independent repeats, *P < 0.05 by *t* test. (D,E) Transwell assay indicated that the migration and invasion ability of cells were enhanced by miR‐876‐5p knockdown in both U2OS and MG63 cells. n = three independent repeats, *P < 0.05 by test

Furthermore, to investigate the functional role of miR‐876‐5p in OS cells, we used lentiviruses to establish miR‐876‐5p stably high‐expressing U2OS and MG63 cells (*P* < 0.05, Figure 2A). The CCK8 and EdU assays showed that miR‐876‐5p restoration inhibited the proliferation of OS cells (*P* < 0.05, Figure 2B,C). Furthermore, the migrated and invaded cells were prominently reduced after miR‐876‐5p overexpression in both U2OS and MG63 cells (*P* < 0.05, Figure 2D,E). Next, the expression of miR‐876‐5p was knocked down by lentivirus‐mediated miR‐876‐5p inhibitors in U2OS and MG63 cells and the knockdown efficiency was verified by qRT‐PCR after 72 hours infection (*P* < 0.05, Figure 3A). Conversely, miR‐876‐5p knockdown significantly promoted the proliferation, migration and invasion of OS cells in vitro (*P* < 0.05, Figure 3B‐D). These data suggest that miR‐876‐5p plays a tumour‐suppressive role in OS.

### 3.3 miR‐876‐5p suppresses the growth of MG63 cells in mice

**Figure 4:**
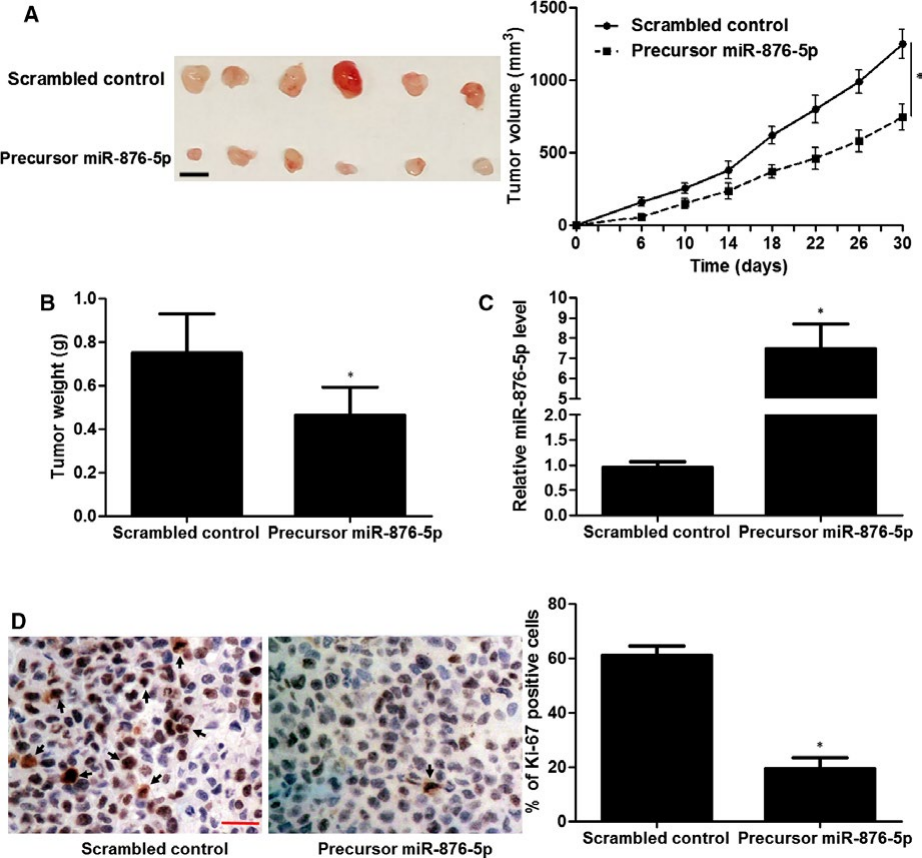
miR‐876‐5p inhibits tumour growth of osteosarcoma (OS) in vivo. A, Left: Photograph of tumours derived from both miR‐876‐5p overexpression group (n = 6) and control group (n = 6). Right: Tumour growth curves indicated that U2OS cells with miR‐876‐5p overexpression grew significantly slower than control cells in mice. Scale bar: 1 cm. *P < 0.05 by ANOVA. B, miR‐876‐5p overexpression significantly reduced tumour weight in experimental group (n = 6) compared to control group (n = 6). *P < 0.05 by *t* test. C, High miR‐876‐5p level was detected in tumour tissues from experimental group (n = 6) compared to that in control group (n = 6). *P < 0.05 by *t* test. D, Immunohistochemical staining of Ki‐67 showed that miR‐876‐5p overexpression inhibited the proliferation of MG63 cells in vivo. Scale bar: 50 μm. n = 6, *P < 0.05 by *t* test

To further confirm the effects of miR‐876‐5p on the proliferation of OS cells in vivo, we established a subcutaneous tumour formation model in nude mice. MG63 cells that were transfected with scrambled control or precursor miR‐876‐5p were subcutaneously injected into the right flank of mice. Thirty days after implantation, the tumour growth curves showed that ectopic expression of miR‐876‐5p significantly inhibited the tumour growth of OS in mice (*P* < 0.05, Figure 4A), and reduced the tumour weight (*P* < 0.05, Figure 4B). The high expression of miR‐876‐5p in tumour tissues was confirmed in miR‐876‐5p overexpression group compared to control group (*P* < 0.05, Figure 4C). Moreover, IHC analysis of the tumour tissues indicated that the percentage of Ki‐67 positive tumour cells in miR‐876‐5p overexpression group was markedly lower than that in control group (*P* < 0.05, Figure 4D).

### 3.4 c‐Met is a target gene of miR‐876‐5p in OS cells

**Figure 5:**
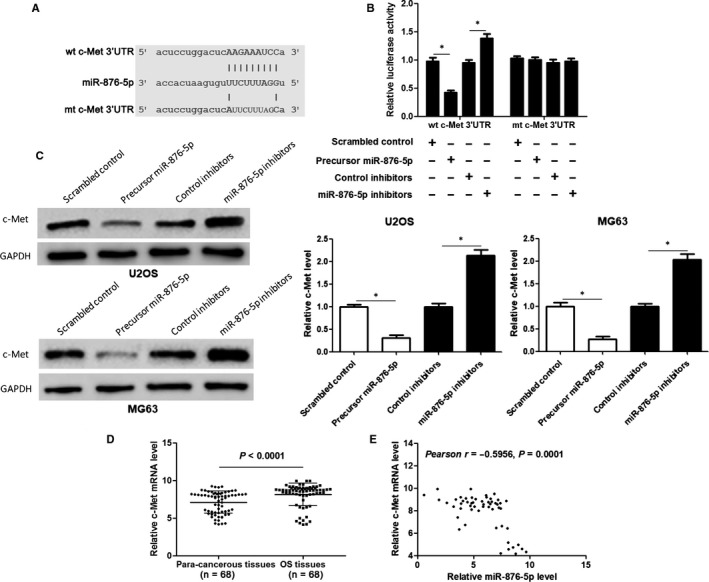
miR‐876‐5p inversely regulates c‐Met expression via posttranscriptional modulation. A, The complementary binding sequences of miR‐876‐5p in wild‐type (wt) 3′UTR of c‐Met. Mutated (mt) 3′UTR of c‐Met was generated to disturb the binding between miR‐876‐5p and c‐Met. B, miR‐876‐5p overexpression obviously inhibited whereas miR‐876‐5p knockdown significantly increased the luciferase activity that carried wt but not mt 3′UTR of c‐Met. n = three independent repeats, *P < 0.05 by *t* test. C, miR‐876‐5p overexpression obviously reduced whereas miR‐876‐5p knockdown significantly increased c‐Met abundance in both U2OS and MG63 cells. n = three independent repeats, *P < 0.05 by *t* test. D, The expression of c‐Met mRNA in OS tissues was significantly higher than that in matched para‐cancerous tissues. n = three independent repeats, *P < 0.05 by *t* test. E, miR‐876‐5p expression was negatively correlated with c‐Met mRNA level in OS tissues. n = 68, *P < 0.05 by Pearson correlation test

To disclose the underlying mechanism involved the tumour‐suppressive role of miR‐876‐5p in OS, potential targets of miR‐876‐5p were predicted by starBase V3.0 online platform. c‐Met, a well‐known oncogene in OS, caught our attention. Then, dual luciferase reporter assay indicated that miR‐876‐5p overexpression significantly reduced while miR‐876‐5p knockdown prominently increased luciferase intensity of vector carrying wt c‐Met 3′UTR in HEK293T cells (*P* < 0.05, Figure 5B). While, modulating miR‐876‐5p level did not alter luciferase intensity of vector carrying mt c‐Met 3′UTR (Figure 5B). Furthermore, miR‐876‐5p restoration significantly reduced while miR‐876‐5p knockdown increased the level of c‐Met protein in both U2OS and MG63 cells (*P* < 0.05, Figure 5C). The expression of c‐Met protein in xenograft tissues from miR‐876‐5p overexpression group was significantly lower than that in control group (*P* < 0.05, Figure S1A). Quantitative real‐time PCR analysis indicated that the expression of c‐Met mRNA in OS tissues was obviously higher than that in para‐cancerous tissues (*P* < 0.0001, Figure 5D). Notably, an inverse correlation between miR‐876‐5p and c‐Met expression was confirmed in OS tissues (*r* = −0.5956, *P* < 0.0001, Figure 5E). The level of c‐Met protein in OS tissues with high miR‐876‐5p expression was obviously lower than that in miR‐876‐5p low‐expressing OS tissues (*P* < 0.05, Figure S1B). These data suggest that miR‐876‐5p directly binds to the 3′UTR of c‐Met to repress its expression.

### 3.5 c‐Met participates in the tumour‐suppressive role of miR‐876‐5p in OS cells

**Figure 6:**
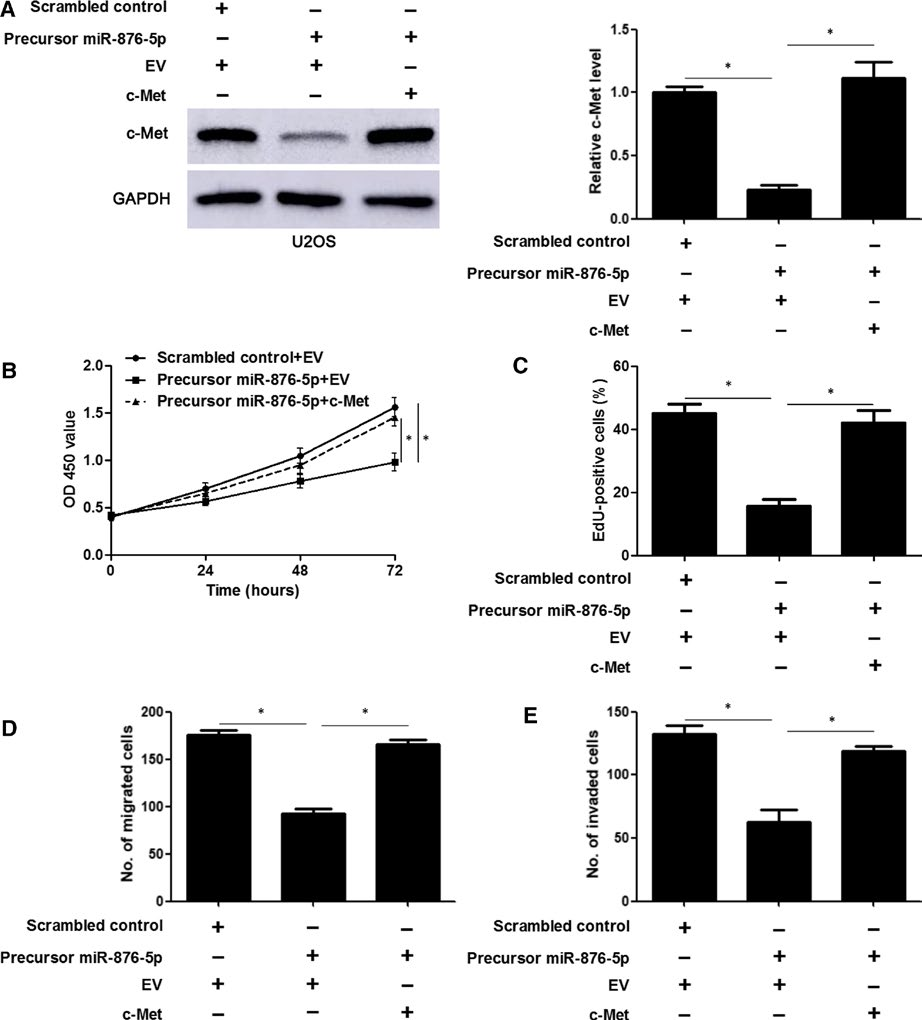
c‐Met rescues miR‐876‐5p attenuated U2OS cell proliferation, migration and invasion. A, U2OS cells were transfected with indicated vectors and detected by immunoblotting for c‐Met expression. B, Cell Counting Kit‐8, (C) 5‐ethynyl‐2'‐deoxyuridine (EdU), and (D,E) Transwell assay were performed to determine the proliferation, migration and invasion of U2OS cells after transfection. n = three independent repeats, *P < 0.05 by ANOVA

To study whether c‐Met was a downstream effector of miR‐876‐5p in OS cells, the expression of c‐Met was resorted via plasmid transfection in miR‐876‐5p overexpressing U2OS cells and confirmed by immunoblotting (*P* < 0.05, Figure 6A). As shown in Figure 6B and C, forced expression of c‐Met reversed the growth arrest of U2OS cells induced by miR‐876‐5p overexpression (*P* < 0.05). Moreover, c‐Met restoration rescued miR‐876‐5p attenuated migration and invasion abilities of U2OS cells (*P* < 0.05, Figure 6D & E). Next, we further confirmed that c‐Met restoration abolished the tumour‐suppressive role of miR‐876‐5p in MG63 cells (*P* < 0.05, Figure S1A‐E). These results demonstrate that miR‐876‐5p inhibits the proliferation, migration and invasion of OS cells through repression of c‐Met.

## 4 DISCUSSION

Accumulating evidence have revealed that miRNAs regulate many oncogenes and tumour suppressors in cells, and lead to initiation and progression of cancer.^21^ Recent years, several miRNAs have been identified as key regulators of OS progression and excellent predictors for clinical outcome of OS patients^22^, ^23^ Decreased expression of miR‐876‐5p has been observed in HNSCC, HCC, ESCC and lung cancer.^14^, ^15^ Moreover, miR‐876‐5p underexpression is associated with poor prognostic features and reduced survival of HCC patients.^16^ In the present study, we investigated the expression of miR‐876‐5p and its clinical significance in OS. Quantitative real‐time PCR data showed that miR‐876‐5p expression was decreased in OS tissues compared with para‐cancerous tissues. Clinical data analysis revealed that OS tissues with advanced clinical stage and poor differentiation had an obvious lower expression of miR‐876‐5p. Moreover, low miR‐876‐5p level predicted poor prognosis of OS patients. These data indicate that miR‐876‐5p may be used as a potential biomarker for diagnosis and prognosis of HCC.

The tumour‐suppressive role of miR‐876‐5p has been reported in HNSCC, HCC, ESCC and lung cancer.^14^, ^15^ Subsequently, miR‐876‐5p was found to inhibit the proliferation, migration and invasion of OS cells, and restrained tumour growth in mice, suggesting a tumour‐suppressive role of miR‐876‐5p. MiRNAs exert their roles by regulating target genes. Several targets of miR‐876‐5p have been indentified in other human cancers. For example, miR‐876‐5p inhibits EMT and tumour metastasis of lung cancer by repressing bone morphogenetic protein 4 (BMP‐4).^14^ MiR‐876‐5p suppresses tumour growth and metastasis of ESCC by targeting Melanoma Antigen Genes‐A (MAGE‐A) family expression.^15^ Furthermore, miR‐876‐5p restrains HCC progression via suppression of BCL6 corepressor like 1 (BCORL1) and DNA methyltransferase 3α (DNMT3A).^16^, ^17^ Thus, the mechanism involved in the functional role of miR‐876‐5p in OS needs to be investigated. We performed starBase V3.0 online platform to predict the candidate targets of miR‐876‐5p. Then, c‐Met was considered as a direct target of miR‐876‐5p in OS. MiR‐876‐5p negatively regulated c‐Met abundance in OS cells and inversely associated with c‐Met mRNA expression in OS tissues. c‐Met, activated MET oncoprotein, has been identified to play a critical role in tumourigenesis and tumour metastasis including OS.^24^ c‐Met is highly expressed in OS tissues and facilitates OS cell proliferation, migration and invasion.^25^, ^26^ Previous studies have reported that c‐Met is regulated by several miRNAs in OS including miR‐613,^27^ miR‐454^28^ and miR‐34a.^29^ Notably, our study reported for the first time that miR‐876‐5p was implicated in the posttranscriptional regulation of c‐Met in OS. More importantly, the inhibition of OS cell proliferation, migration and invasion induced by miR‐876‐5p overexpression was attenuated by restoration of c‐Met in OS cells. These results manifest that miR‐876‐5p mainly relies on suppression of c‐Met to inhibit OS cell proliferation and metastasis.

Collectively, our findings demonstrate that miR‐876‐5p is down‐regulated in OS tissue samples. Low miR‐876‐5p level associates with malignant clinical features and poor prognosis. In addition, miR‐876‐5p inhibits OS cell proliferation, migration and invasion processes. c‐Met is a direct downstream target and functional effector, which mediates the tumour‐suppressive role of miR‐876‐5p in OS cells. These findings suggest that miR‐876‐5p is a potential clinical therapeutic target in OS.

## 5 CONCLUSIONS

To conclude, we investigated the expression of miR‐876‐5p between OS and matched para‐cancerous tissues, and elucidated the functional role of miR‐876‐5p and its underlying molecular mechanisms in OS cells. Our study revealed that underexpression of miR‐876‐5p was a frequent event in OS tissues and indicated poor prognosis of patients. Functionally, miR‐876‐5p exerted a tumour‐suppressive role by inhibition of OS cell proliferation, migration and invasion via suppression of c‐Met. Our results may provide a novel theoretical and experimental basis for the pathogenesis of OS, and identify novel treatment targets.

## CONFLICTS OF INTEREST

All authors declare no conflicts of interest.
