# Supplementary figures and images for: MicroRNA‐876‐5p inhibits cell proliferation, migration and invasion by targeting c‐Met in osteosarcoma
Source: J Cell Mol Med. 2019 Feb 17;23(5):3293–301. doi: 10.1111/jcmm.14217 (PMC6484334; doi:10.1111/jcmm.14217)

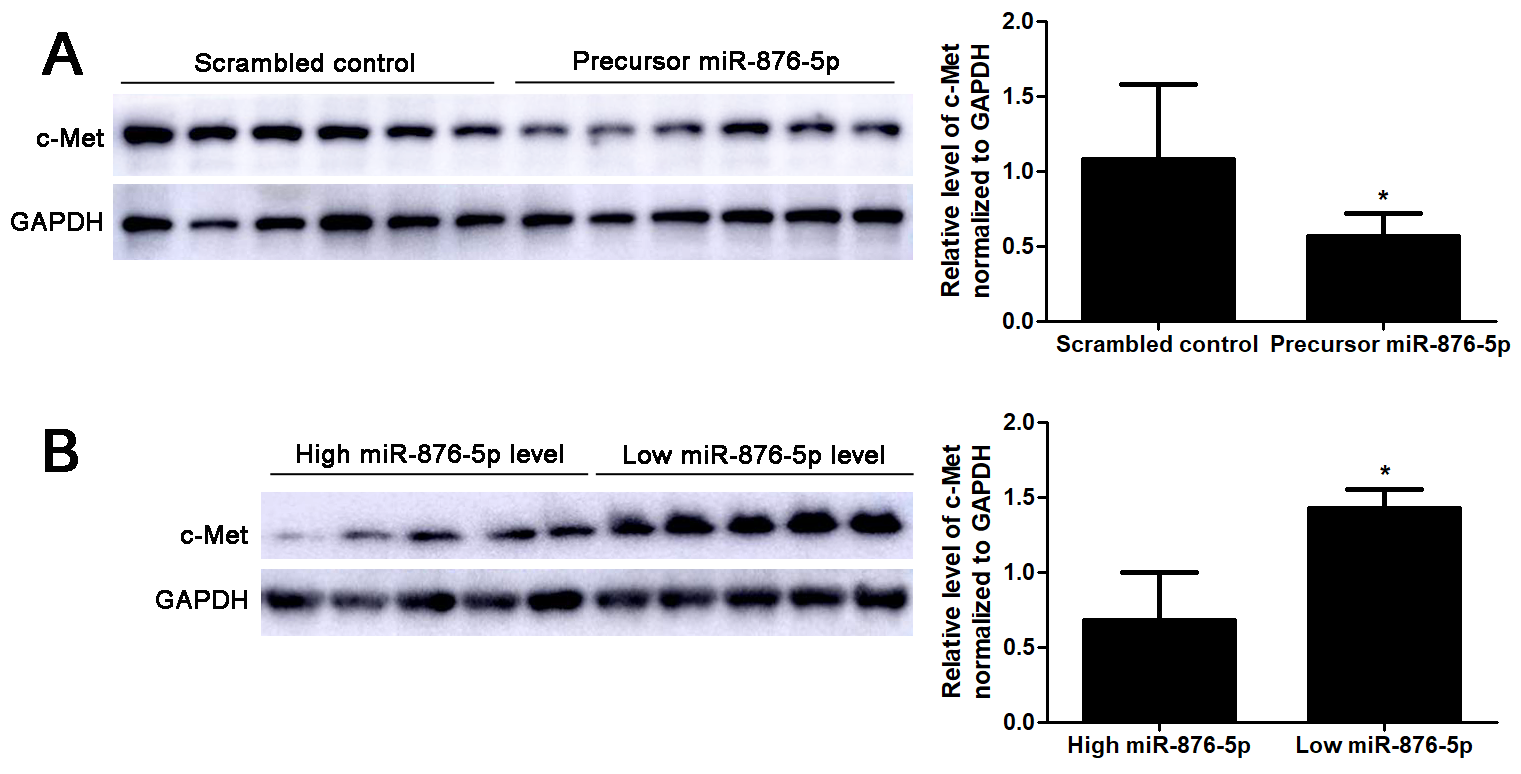

Supplement: Supplementary file 1 [file JCMM-23-3293-s001.tif]

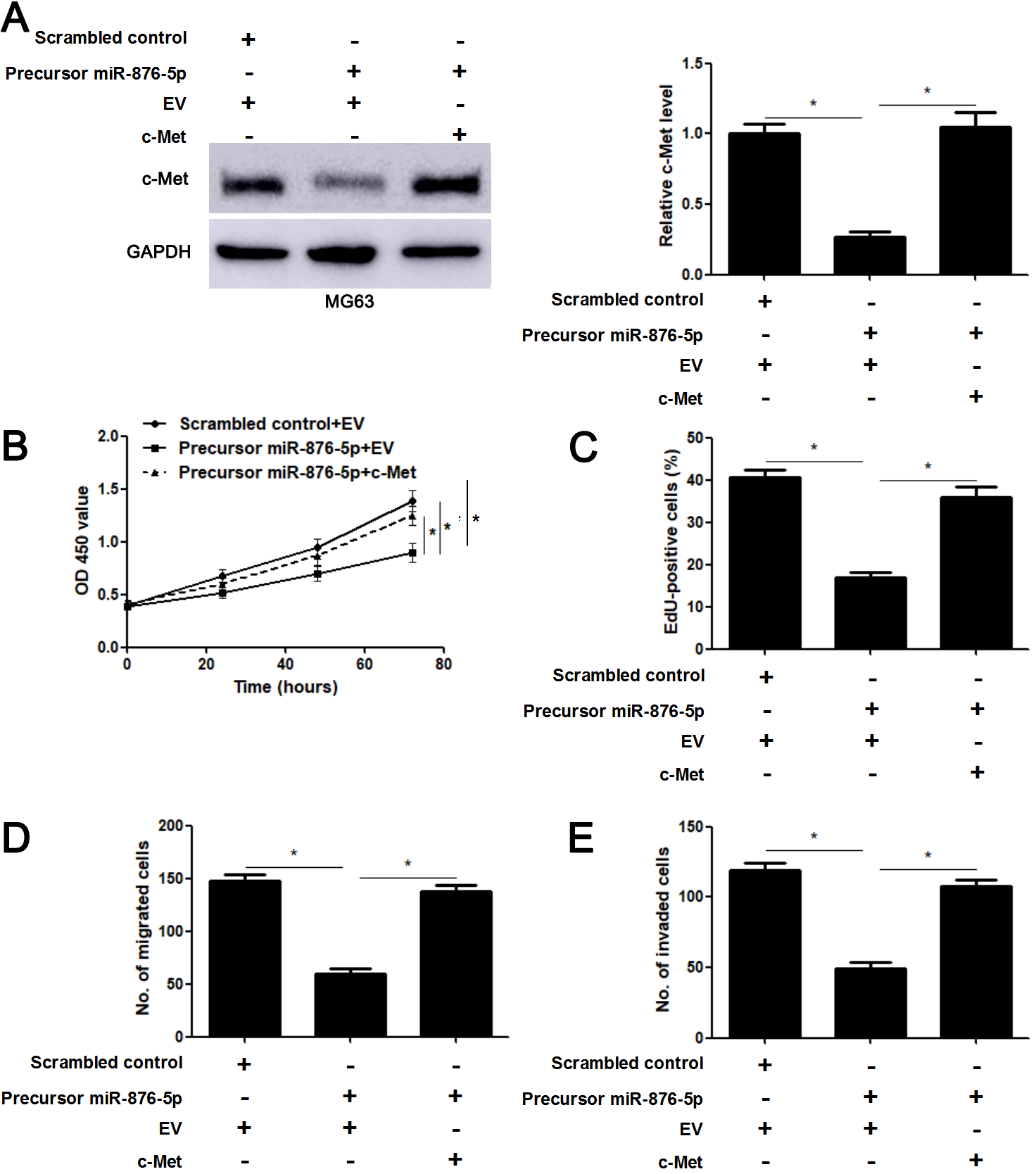

Supplement: Supplementary file 2 [file JCMM-23-3293-s002.tif]
