# Supplementary material for: MicroRNA‐876‐5p inhibits cell proliferation, migration and invasion by targeting c‐Met in osteosarcoma
Source: J Cell Mol Med. 2019 Feb 17;23(5):3293–301. doi: 10.1111/jcmm.14217 (PMC6484334; doi:10.1111/jcmm.14217)
Supplement: Supplementary file 3 [file JCMM-23-3293-s003.docx]

**Supplementary Table 1**. List of primers used in this paper

| **Gene** | **Primer** | **Sequence(5′-3′)** |
| --- | --- | --- |
| miR-876-5p | forward | ACUUAAUGAAACAUUUGGUGGU |
|  | reverse | TGUUTTUCTTTGTUUUCCUCCU |
| c-Met | forward | GAGGCAGTGCAGCATGTAGT |
|  | reverse | GATGATTCCCTCGGTCAGAA |
| U6 | forward | GCTTCGGCAGCACATATACTAAAAT |
|  | reverse | CGCTTCACGAATTTGCGTGTCAT |
| GAPDH | forward | CCATGTTCGTCATGGGTGTG |
|  | reverse | GGTGCTAAGCAGTTGGTGGTG |

**Supplementary Figure 1 miR-876-5p inversely correlated with c-Met expression in OS.** (A) Immunoblotting analysis indicated that the expression of c-Met protein in xenograft tissues from miR-876-5p overexpression group (n = 6) was significantly lower than that in control group (n = 6). **P* < 0.05 by t test. (B) The expression of c-Met protein in OS tissues with high miR-876-5p level (n = 5) was obviously lower than that in cases with low miR-876-5p level (n = 5). **P* < 0.05 by t test.

**Supplementary Figure 2 c-Met rescues miR-876-5p attenuated MG63 cell proliferation, migration and invasion.** (A) MG63 cells were transfected with indicated vectors and detected by immunoblotting for c-Met expression. (B) CCK-8, (C) EdU, and (D and E) Transwell assay were performed to determine the proliferation, migration and invasion of MG63 cells after transfection. n = three independent repeats, **P* < 0.05 by ANOVA.
